# Pumping Electron-Positron Pairs from a Well Potential

**DOI:** 10.1038/srep25292

**Published:** 2016-04-29

**Authors:** Qiang Wang, Jie Liu, Li-bin Fu

**Affiliations:** 1 National Laboratory of Science and Technology on Computational Physics, Institute of Applied Physics and Computational Mathematics, Beijing 100088, China.; 2 HEDPS, Center for Applied Physics and Technology, Peking University, Beijing 100871, China; 3 CICIFSA MoE College of Engineering, Peking University, Beijing 100871, China

## Abstract

In the presence of very deep well potential, electrons will spontaneously occupy the empty embedded bound states and electron-positron pairs are created by means of a non-perturbative tunneling process. In this work, by slowly oscillating the width or depth, the population transfer channels are opened and closed periodically. We find and clearly show that by the non-synchronous ejections of particles, the saturation of pair number in a static super-critical well can be broken, and electrons and positrons can be pumped inexhaustibly from vacuum with a constant production rate. In the adiabatic limit, final pair number after a single cycle has quantized values as a function of the upper boundary of the oscillating, and the critical upper boundaries indicate the diving points of the bound states.

In a static, uniform and very strong electric field, the QED vacuum may break down and decay into electron-positron pairs due to a quantum tunneling effect^1^,^2^,^3^. Time dependent fields can also generate pairs through another mechanism, that electrons in Dirac Sea transit into positive states via photon absorption^4^,^5^,^6^. Positron beam is a nondestructive probe in positron annihilation spectroscopy for the study of atomic-scale structure of materials^7^,^8^. Pair creation is an important issue in the study of laser-vacuum, laser-matter interaction, and also in astrophysics since it is thought to be associated with the supernova explosion^9^. In laboratory, pairs have been generated by the collision of heavy ions^10^ and the collision of an intense laser pulse and a 46 GeV electron beam^11^. Recently, MeV positron beam with high density was obtained through laser-accelerated electrons irradiating high-Z solid targets^12^. However, due to the presently unfeasible Schwinger critical field strength, which is about 10^16^ *V*/*cm* and correspond to a laser intensity of about 10^29^ *W*/*cm*^2^, pairs created from pure laser light has not been observed yet. In light of the rapid advance of laser technology a good theoretical understanding of the pair creation in strong laser fields becomes highly desirable^13^.

For a well potential of depth *V*_0_, if *V*_0 _> 2*c*^2^, the domain *c*^2^−*V*_0_ < *E* < −*c*^2^ exist. Bound states in the well may join continuum waves of the same energy *E* < −*c*^2^ out the well and their wave functions have non-zero probability outside. So electrons from the filled Dirac Sea will spontaneously occupy these empty bound states. The holes left (identified as positrons) will travel away from the edges of the well to infinity^14^. This is the picture of spontaneous creation of electron-positron pairs. For a static well potential, electrons will fill the embedded bound states, and the Pauli principle will prevent further pair creation, resulting an asymptotic saturation behavior^15^,^16^,^17^. The number of pairs created should be the number of bound states which meet these conditions.

If the potential is time dependent, the situation is more complicated. In paper^16^ by varying the width of potential, the effects of open and close a pair-creation channel (embedded bound state) were studied. After enough time for saturation, the pair number will increase if one more channel is opened, but will not decrease if one of the two channels is closed. The reason is that the annihilation needs the electron and positron to be in the same place, which is not satisfied because the electrons remain in the well while the positrons have left the creation zone and escaped to the opposite direction. Naturally, one would wonder that if the channel is opened and closed periodically, can this mechanism lead to a continuously pair creation? Moreover, for fixed width and varying depth, since the behaviors of energy spectra are similar, will something similar happen? Motivated by these questions, in this work we examine the pair creation in a well potential with its width or depth oscillating. By oscillating the width or depth, the electrons confined in the well will be released and the the saturation of pair number will be broken. We find that this can lead to a constant production rate, which means that pairs can be pumped inexhaustibly from the well.

The paper is organized as follows. First, we present our model. The well potential is set to be oscillating in two modes, the width oscillating mode and the depth oscillating mode. The energy spectra are shown as a function of the width or depth. Then in both two modes, the time evolution of pair number, spacial density and pumping rate are studied. Furthermore, we investigate the adiabatic limit of the oscillating. Brief summary and discussion are provided next. The numerical method we employed follows in the last.

## Model: one-dimensional well potential with oscillating width or depth

The well potential is defined by two Sauter potentials^1^, which represent two localized electric fields that have identical intensities and frequencies, but phases differ by a shift of *π*,


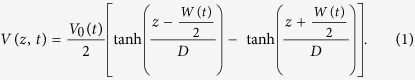


*D* is the width of the potential edge (a measure of the width of the electric field), and we set *D* = 0.3*λ*_*C*_. (Here and below we use atomic units [a.u.], *m* = *ħ* = *e* = 1, *c* = 137.036, Compton wavelength *λ*_*C*_ = 1/*c*). *W*(*t*) is the potential width (the separation between two localized electric fields). The numerical box size is *L* = 2.5 (in [a.u.], omitted in the following). We define the two modes as: (1) **W-oscillating mode**: with *V*_0_ = 2.53*c*^2^ constant, 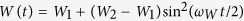; (2) **V-oscillating mode**: with *W* = 10*λ*_*C*_ constant, 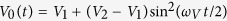. In this paper we assume *W*_1_ = 0 and *V*_1_ = 0, then *W*(*t*) (or *V*_0_(*t*)) varies as a sine function between zero and its upper boundary *W*_2_ (or *V*_2_).

**Figure 1:**
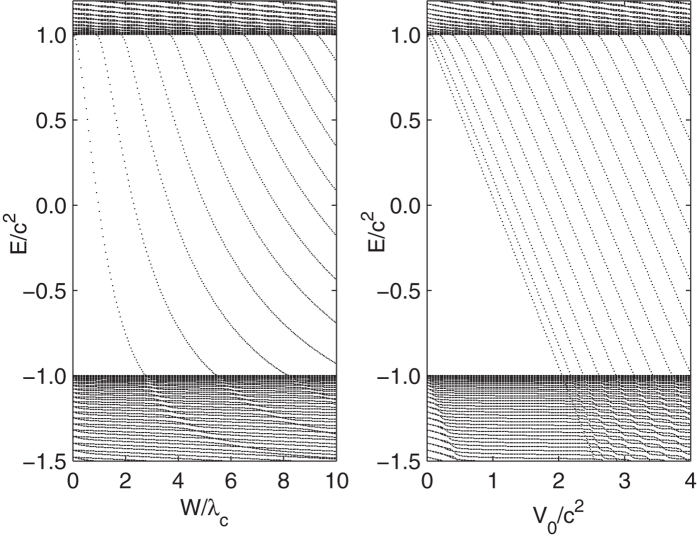
Energy spectrum as a function of the width or the depth of the potential. (a) *V*_0_ = 2.53*c*^2^, as *W* increasing, the bound states dive into the Dirac Sea at *W* = 2.79, 5.51, 8.21... (in units of *λ*_*C*_). (b) *W* = 10*λ*_*C*_, as *V*_0_ increasing, the bound states dive into the Dirac Sea at *V*_0_ = 2.05, 2.19, 2.38, 2.62, 2.87, 3.15, 3.43, 3.73, ... (in units of *c*^2^).

The Dirac Hamiltonian of this system is (it is sufficient to focus on only the spin-less state)


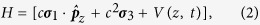


where ***σ***_1_, ***σ***_3_ are Pauli matrices. Numerical energy spectra of the Hamiltonian are presented in Fig. 1 for the two modes. The behaviors of the bound states diving into the negative continuum, and the accompanied critical widths or depths are illustrated. For example, if *V*_0_ = 2.53*c*^2^, there are bound states embedded only when *W* > 2.79*λ*_*C*_.

## Results

### Time evolution of pair number

**Figure 2:**
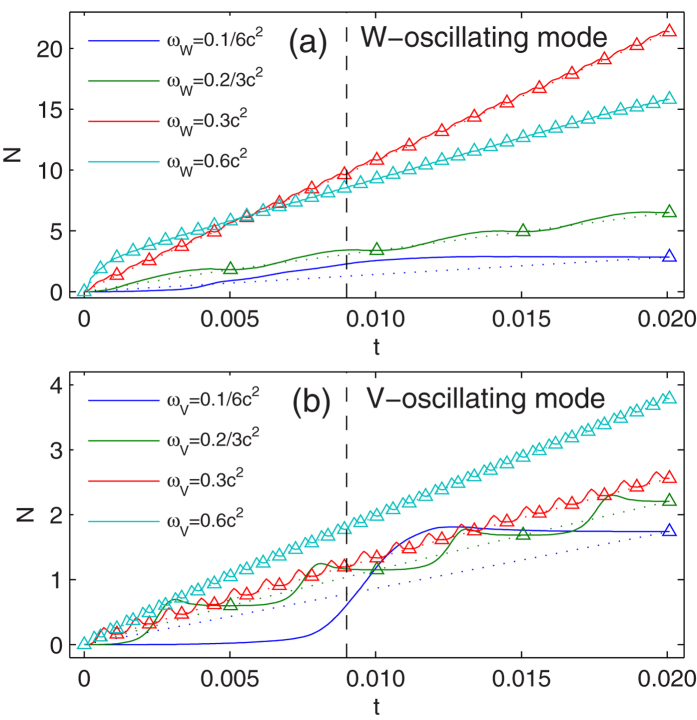
The time evolution of the total number of pairs for both W-oscillating and V-oscillating modes. (**a**) W-oscillating mode, *V*_0_ = 2.53*c*^2^, *W*_2_ = 10*λ*_*C*_; (**b**) V-oscillating mode, *W* = 10*λ*_*C*_, *V*_2_ = 2.53*c*^2^. The triangles denote pair numbers when the field is absent. The dot lines just link these triangles. The dash line represent *t* = 0.009 when positrons arrive the boundary, *z* = ±*L*/2 = ±1.25.

We graph the time evolution of the pair number *N*(*t*) defined in equation (14) for both W-oscillating and V-oscillating modes in Fig. 2. The width frequency *ω*_*W*_ and depth frequency *ω*_*V*_ are assumed to be relative low comparing to the gap 2*c*^2^, so that the photon absorption mechanism is not remarkable. The total time is 120*π*/*c*^2^ ≈ 0.02. The dash lines indicate the time *t* ≈ *L*/(2*c*) ≈ 0.009 when the particles arrive the boundary, *z* = ±*L*/2 = ±1.25. Since *W*_1_ = 0 and *V*_1_ = 0, if the time is an integer multiples of the period (*T*_*W*_ or *T*_*V*_), denoted by the triangles in Fig. 2, the system Hamiltonian degenerates to a field free one.

### W-oscillating mode

In Fig. 2(a), we illustrate the pair number *N*(*t*) as a function of time for *ω*_*W*_ = 0.1/6*c*^2^, 0.2/3*c*^2^, 0.3*c*^2^, 0.6*c*^2^. The depth *V*_0_ is fixed at *V*_0_ = 2.53*c*^2^ as Fig. 1(a). The lower and upper boundaries of width are *W*_1_ = 0 and *W*_2_ = 10*λ*_*C*_, corresponding zero and three bound states embedded respectively. When *W* = *W*_2_, there are also eight bound states exist in the gap −*c*^2^ < *E* < *c*^2^, which can be associated with the pair creation^15^.

**Figure 3:**
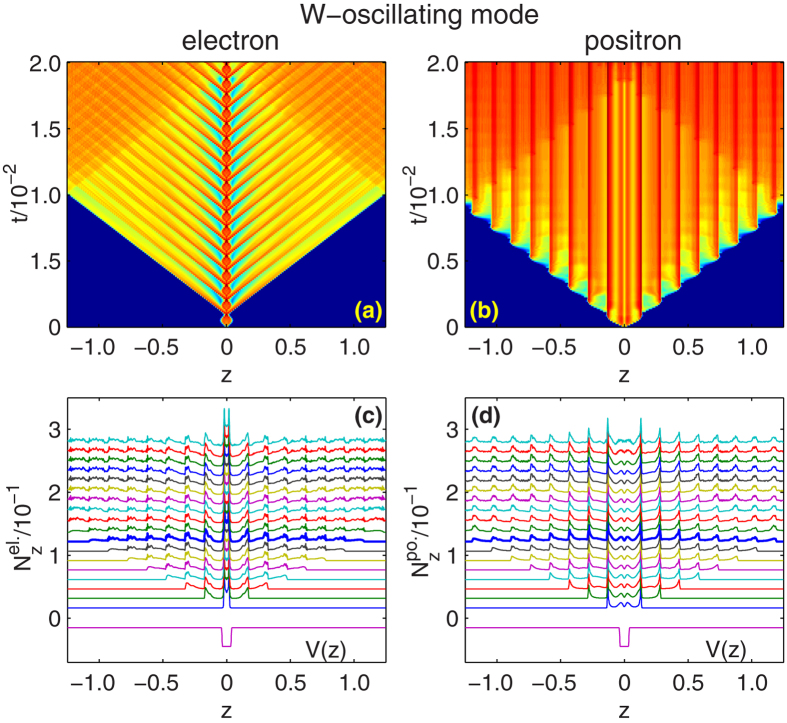
For W-oscillating mode, *ω*_*W*_ = 0.3*c*^2^, the three dimensional diagrams for entire time and the waterfall figures for field free moments (indicated by triangles on curve *ω*_*W*_ = 0.3 in Fig. 2(a)), for electron spacial density (**a**,**c**) and positron spacial density (**b**,**d**). The thicker curves in sub-figure (**c**,**d**) mark the last cycles before positrons arrive the boundary. The well potentials *V*(*z*) with *V*_0_ = 2.53*c*^2^ and *W* = 10*λ*_*C*_ are included on the bottom for comparison. All other parameters are the same as Fig. 2(a).

When *ω*_*W*_ = 0.1/6*c*^2^, the width *W* can only finish one cycle in the total time 120*π*/*c*^2^. *N*(*t*) begin to arise before the first bound state dives into the negative continuum when *W*(*t*) = 2.79*λ*_*C*_ and *t* = 3.57 × 10^−3^. The reason is the non-adiabatic varying width. *N*(*t*) will begin to arise precisely at the time when *W*(*t*) = 2.79*λ*_*C*_ in the adiabatic case (*ω*_*W*_ → 0, see the discussion below). *N*(*t*) increases as more bound states dive in, and reaches its maximum *N* = 2.89 at *t* = 1.37 × 10^−2^, between *t* = 1.28 × 10^−2^ and 1.47 × 10^−2^, at which time the third and the second bound state were pulled out the Dirac Sea. Undergoing the particle-antiparticle annihilation, *N*(*t*) decreases but remains an appreciable value *N* = 2.85 in the end. In the latter half of this cycle, the embedded bound states depart from the Dirac Sea, return to the positive continuum, and become scattering states. The released positrons are reflected by the numerical box boundary, come back to the interaction region and affect the pair generation after. Though the effect is weak when *ω*_*W*_ = 0.1/6*c*^2^, it is non-ignorable when, i.e., *ω*_*W*_ = 0.3*c*^2^ (see Fig. 3 for details).

For *ω*_*W*_ = 0.2/3*c*^2^ and *ω*_*W*_ = 0.3*c*^2^, *W* can finish four and eighteen cycles in the total time and the pair number are *N* = 6.49, *N* = 21.4 in the end. For *t* < 0.009, *W* can finish one and eight cycles respectively. In each cycle, the positrons are repulsed by the electric field to the infinity once they were generated, while the electrons are limited in the well when the field is strong enough and extruded out as the well is turning off, avoiding the inevitable Pauli block in the non-varied static well. The non-synchronous ejections prevent the annihilation and lead to a high production rate.

Every next cycle starts from field free and is independent on the previous cycle. In Fig. 2, the dot lines link the triangles which denote the pair number when the field is absent. We can find that the pair generation before *t* = 0.009 denoted by the dot lines is linearly depend on time for low frequency *ω*_*W*_. If the system length *L* is infinite and there is no reflection at the boundary, the pairs can be pumped inexhaustibly with a constant production rate from the well. Even for *ω*_*W*_ = 0.6*c*^2^, there is nonlinear effect at the beginning, the generation rate becomes stable soon.

Due to the finite period *T*_*W*_ and the bound states in the gap, particle generation and ejection processes are not monotonic with the increase of the frequency *ω*_*W*_. However, ignoring the reflection, if *ω*_*W*_ is very small, we can expect a linear dependent of final pair number on the frequency *ω*_*W*_.

### V-oscillating mode

The number of pairs *N*(*t*) as a function of time are presented in Fig. 2(b) for *ω*_*V*_ = 0.1/6*c*^2^, 0.2/3*c*^2^, 0.3*c*^2^ and 0.6*c*^2^. As in Fig. 1(b), the width *W* is fixed at *W* = 10*λ*_*C*_, while the depth varies between *V*_1_ = 0 and *V*_2_ = 2.53*c*^2^, corresponding zero and three bound states embedded respectively.

For *ω*_*W*_ = 0.1/6*c*^2^, the first bound state dives in at *t* = 7.20 × 10^−3^, at which time there are already *N* = 8.83 × 10^−2^ pair generated. The first bound state departs the negative continuum after the third and the second ones, at *t* = 1.29 × 10^−2^, when *N*(*t*) reach its maximum *N* = 1.81. Finally, there are *N* = 1.74 pairs survived at *t* = 120*π*/*c*^2^. For *ω*_*V*_ = 0.2/3*c*^2^, 0.3*c*^2^, 0.6*c*^2^, the pair number in the end are *N* = 2.21, 2.56, 3.78.

Instead of pulling and pushing the walls of the well in W-oscillating mode, in this mode it is the rising and falling bottom of the well that control the bound states diving in and departing from the negative continuum. It is also the non-synchronous ejection of the positrons and electrons which dominates the pumping process.

The dot lines here indicate a linear relation between the pair number and time. The final number is not monotonic depending on the frequency *ω*_*V*_, but we can also expect a linear dependent of final pair number on *ω*_*V*_ when *ω*_*V*_ is very small.

Note that although the two modes have the same beginning and ending parameters, the generation rate in the W-oscillating mode is much higher.

### Time evolution of spacial density

To show the pumping process explicitly, we compute the time evolution of spacial density of electrons and positrons (equation (15) and equation (16)) for *ω*_*W*_ = 0.3*c*^2^ and *ω*_*V*_ = 0.3*c*^2^ respectively.

**Figure 4:**
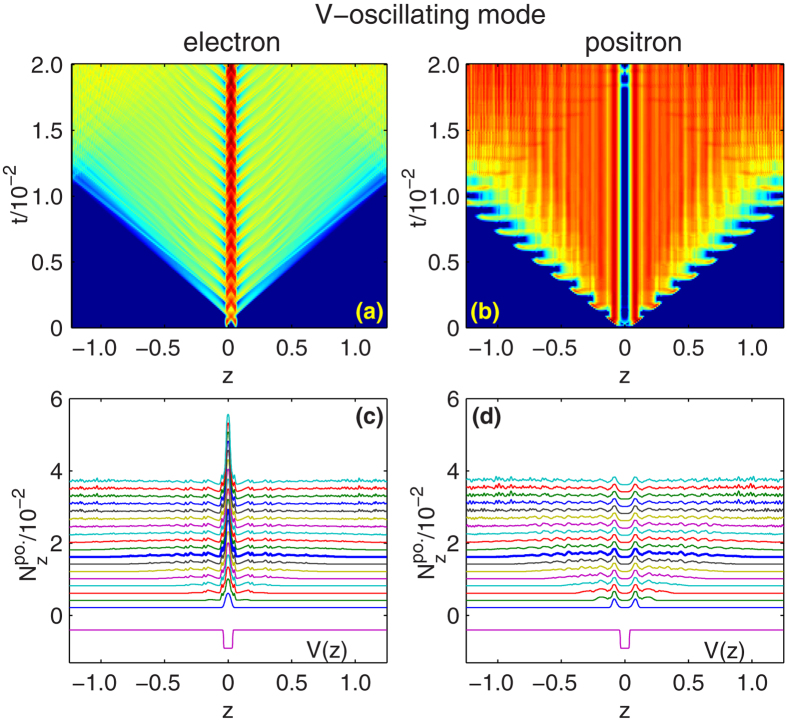
For V-oscillating mode, *ω*_*V*_ = 0.3*c*^2^, the three dimensional diagrams for entire time and the waterfall figures for field free moments (indicated by triangles on curve *ω*_*V*_ = 0.3 in Fig. 2(b)), for electron spacial density (**a**,**c**) and positron spacial density (**b**,**d**). The thicker curves in sub-figure (**c**,**d**) mark the last cycles before positrons arrive the boundary. The well potentials *V*(*z*) with *V*_0_ = 2.53*c*^2^ and *W* = 10*λ*_*C*_ are included on the bottom for comparison. All other parameters are the same as Fig. 2(b).

In Fig. 3, for W-oscillating mode, *ω*_*W*_ = 0.3*c*^2^, we plot the the time evolution of spacial density of electrons and positrons (sub-figure (a) and (b)). Specially, for the moments when the fields are zero, these quantities are plotted in the waterfall figures, Fig. 3(c,d). For V-oscillating mode, *ω*_*V*_ = 0.3*c*^2^, similar diagrams are presented in Fig. 4. For comparison, the well potential *V*(*z*) with wide and depth equal to the upper boundary of the two modes, *W* = *W*_2_ = 10*λ*_*C*_, *V*_0_ = *V*_2_ = 2.53*c*^2^, are included on the bottom. These figures clearly show how the particles are pumped from the well and spread in the numerical box.

Since *ω*_*W*_ = 0.3*c*^2^, the period of the width oscillating is *T*_*W*_ = 1.12 × 10^−3^. Before positrons reach the boundaries, the width can finish eight cycles. If we detect the particle population at the boundary, we can find that positrons arrive the boundary first, at *t* = 9.15 × 10^−3^, in conformity to the estimation *L*/(2*c*) = 9.12 × 10^−3^. Electrons arrive the boundary at *t* = 1.02 × 10^−2^, about one period (*T*_*W*_ or *T*_*V*_) later than the positrons. We can see that the particles reflected by the boundary come back to the interaction region, and cause non-ignorable effect, for example, the non-linearity of the last three triangles in the dot line in Fig. 2(a), *ω*_*W*_ = 0.3*c*^2^.

Comparing with the rising and falling bottom of the well, more work is done by the wall of the well in the case of opening and closing the well. In the W-oscillating mode, the wavefront of the particles are more abrupt and regular. In energy space, higher energy modes are excited, and the spectrum show periodic structure with 0.3*c*^2^ between each peak. In the V-oscillating mode, electrons are lifted and released naturally. Less work is done and only low momenta are excited.

### Time dependent pumping rate

**Figure 5:**
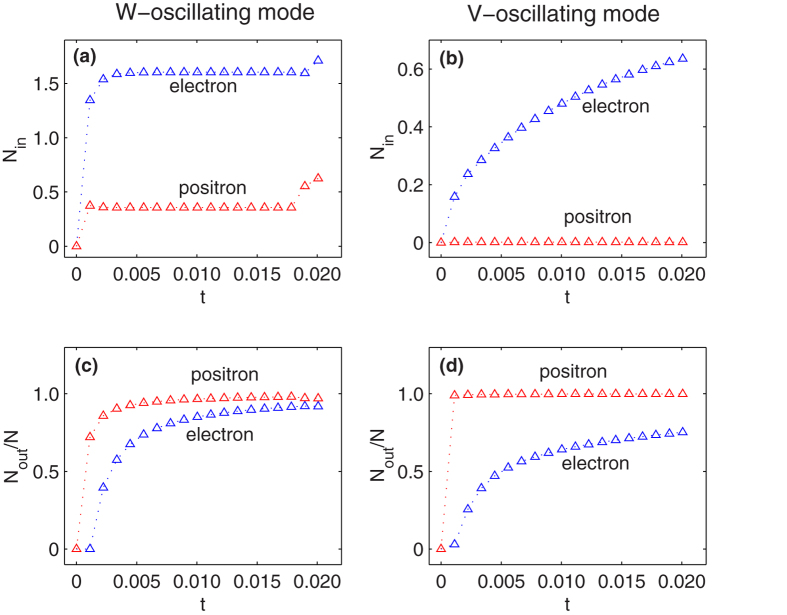
For W-oscillating mode (*ω*_*W*_ = 0.3*c*^2^, sub-figure (**a**,**c**)) and V-oscillating mode (*ω*_*V*_ = 0.3*c*^2^, sub-figure (**b**,**d**)), particles in the well (*N*_*in*_) and the pumping rate *N*_*out*_/*N* are shown as a function of time. The triangles denote the moments when field are absent and the dot lines link them. The blue triangles denote electron and the red denote positron. All parameters are the same as Figs 3 and 4, respectively.

It turns out that in the V-oscillating mode electrons are more inclined to gather in the well region (defined as −5*λ*_*C*_ < *z* < 5*λ*_*C*_) than it in W-oscillating mode. We can integrate the spacial density *N*_*z*_ in this region and get the particle number in the well, 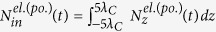. For the pumping process in last section, 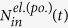 are graphed in Fig. 5(a,b). In W-oscillating mode, as time increasing, 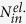 saturates to a constant 1.60 quickly, while 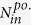 to a constant 0.36. But in V-oscillating mode, 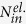 keeps increasing while 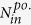 keeps zero. The reason is that in W-oscillating mode positrons can be generated in the well region, while in V-oscillating mode the walls (the electric fields) shut the door upon positrons.

In a pumping process, the pumping rate is vitally important and can be defined as *α*(*t*) = *N*_*out*_(*t*)/*N*(*t*), where *N*_*out*_ = *N* − *N*_*in*_, as shown in Fig. 5(c,d). In both modes, at the end of the first cycle, when *t* = *T*_*W*_ or *T*_*V*_, nearly all the electrons are limited in the well region, while positrons are ejected. In V-oscillating mode, since all the generated positrons are kept out of the well, the pump rate become 1 directly. For electrons in the V-oscillating mode, or electrons and positrons in W-oscillating mode, in the long time limit, *α*(*t*) come to 1 in the form 1−*β*/*t*, where *β* depends on the saturation number of particles in the well and the number of particles can be generated in each cycle.

### The adiabatic limit

**Figure 6:**
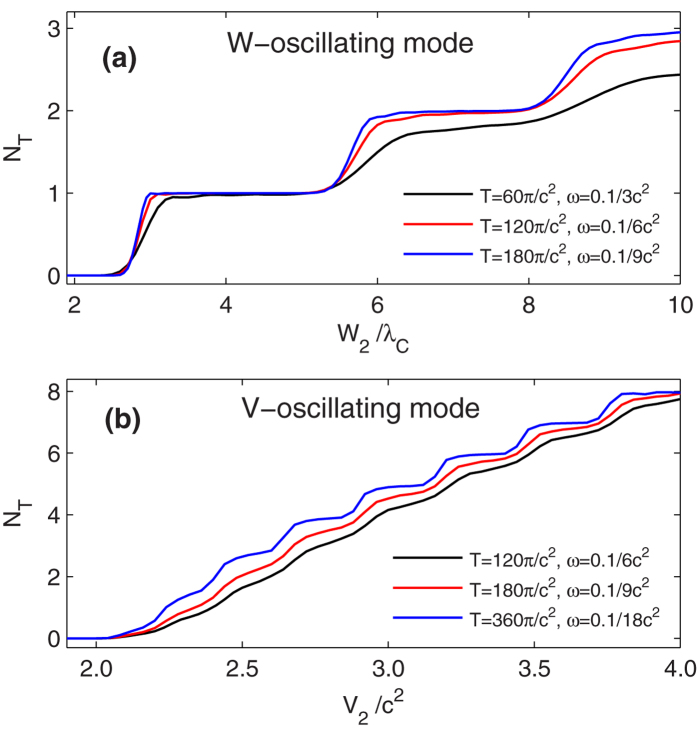
The final pair number after one cycle as a function of the upper boundary of the oscillating width and depth. (**a**) W-oscillating mode, *V*_0_ = 2.53*c*^2^; (**b**) V-oscillating mode, *W* = 10*λ*_*C*_. The total time *T* is chosen equal to one oscillating period.

In Fig. 2, for *ω*_*W*_ = 0.1/6*c*^2^ and *ω*_*V*_ = 0.1/6*c*^2^, there are *N* = 2.85 and *N* = 1.74 pairs survived in the end. We have proposed that in low frequency limit, the pairs survived finally should equal to three, the maximum number of embedded bound states swept in one cycle of each mode. In Fig. 6, ignoring the reflection, for each frequency, the total time is chosen equal to the period for both modes, so that the oscillation can only finish one cycle. The survived final pair number *N*_*T*_ as a function of the upper boundary of the oscillating width (*W*_2_) and depth (*V*_2_) are presented.

In the adiabatic limit, a sub-critical well potential cannot trigger pairs. As the width or depth increasing, the bound states in the gap dive into the negative continuum successively. Pairs can be generated and saturated to the number of embedded bound states. However, as the width and depth decreasing, bound states depart the negative continuum successively and the generated pairs cannot annihilate because of the non-synchronous ejection. Finally, the number of pairs survived at the end of this cycle has quantized values, equal to the maximum number of bound states embedded. The quantized values depend on the upper boundary of the two oscillating cycle (*W*_2_ or *V*_2_).

In Fig. 6, the curves of *N*_*T*_ vs. *W*_2_ or *V*_2_ are like a flight of stairs. As the frequency become lower, the rising edges of the stairs become more sharper. In the limit *ω*_*W*_, *ω*_*V*_ → 0, the the rising edge of the stairs will precisely locate at the points where the bound states dive into the negative continuum. These points are *W* = 2.79, 5.51, 8.21... (in units of *λ*_*C*_), and *V*_0_ = 2.05, 2.19, 2.38, 2.62, 2.87, 3.15, 3.43, 3.73, ... (in units of *c*^2^), as illustrated in Fig. 1.

The gaps between bound states with −*c*^2^ < *E* < *c*^2^ in V-oscillating mode are smaller than that in W-oscillating mode. To achieve a quasi-adiabatic (finite *T*_*W*_ or *T*_*V*_) simulation, *T*_*V*_ should be larger than *T*_*W*_ to build a similar stairs.

Now, if the quasi-adiabatic oscillating cycle repeat periodically, we can expect a linear increasing pair number, i.e., for Fig. 6(a), *W*_2_ = 7*λ*_*C*_, the final pair number will be 2 times the number of the cycles.

## Discussion

In this work, we have constructed a toy model, in which oscillating width or depth are proposed to break the Pauli block which is a barrier in further pair generation process. Since the bound states diving behaviors in the energy spectra are similar when sweeping the depth or width, the physical process have common points in these two modes. We find that by open and close the transfer channels for population alternately, the non-synchronous ejection of particles prevent the particle annihilation, break the saturation of pair number in a static super-critical well potential, and lead to a high constant production rate. The width oscillating mode can deliver more energy to particles and is more efficient in pumping pairs than the depth oscillating mode. The time evolution of spacial density provide clearly graphical representations for the pumping and the spreading of electrons and positrons. In a quasi-adiabatic case, the final pair number as a function of upper boundary of the oscillating changes abruptly at the diving points of the bound states. This can also be expected to detect the energy structure of a complicated potential.

In order to reduce the computing cost, we have neglected the larger part of the discrete momenta. On the other hand, with the same computing resource, the number of spatial points can be larger to describe the details of the potential. Although the simulation here is done on a personal stand-alone computer, it can be paralleled easily since the time evolution of each negative eigenstate can be done on a single CPU. Furthermore, if the spatial derivative is done by finite difference approximations instead of Fourier transformation here^18^ larger one-dimensional, even two-dimensional system can be simulated by paralleling the algorithm on memory shared parallel computers.

### Method: the numerical quantum field theory approach

Various numerical approaches were developed recently to cope with the pair creation problem which in general is non-equilibrium, non-perturbation, and space-time dependent. For example, the semi classical WKB methods^19^,^20^,^21^, the world-line formalism (string-inspired formalism)^22^,^23^ and the quantum kinetic theory (QKT)^24^,^25^ by solving the quantum Vlasov equation. In this paper we employ the numerical approach to quantum field theory which has been introduced recently to study the pair creation process with full space-time resolution (for a review see^26^). This approach can provide details of the boson^27^,^28^,^29^ or fermion^30^,^31^,^32^ pair creation dynamics, and has been used to research various conceptual problems where the negative energy states must be taken into account, such as the Zitterbewegung^33^, the relativistic localization problem^31^, and the Klein paradox^29^,^34^.

In this approach, the problem is reduced to single particle quantum mechanics formulation. In quantum field theory, the time evolution of the field operator 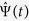 fulfills the Heisenberg equation of motion 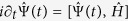. It was proved^30^ that 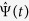 can be obtained equivalently as a solution of the Dirac equation 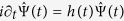, with Hamiltonian *h*(*t*) = *c**α*** ⋅ (***p*** − *eA*/*c*) + *c*^2^*β* + *eV*(***r***, *t*). This equivalence between the quantum field theoretic treatment and the solution of the Dirac equation has also been established in the context of pair creation in heavy-ion collisions^35^,^36^. Thus, the dynamics of 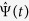 can be obtained via the time evolution of the single particle Dirac equation with space and time taking into account.

This approach can visualize the processes inside the interaction zone, while the traditional scattering matrix approach^14^ which is based on the initial state and the final state of a physical system undergoing a scattering process and cannot offer details inside the interaction region. It does not include the interaction between particles and the back reaction onto the electrodynamic field. To overcome this weakness is beyond the computing power presently. Despite that, in contrast to the quantum kinetic theory which can include the particle collisions and back reaction, but is a mean field approximation and works only for spatial homogeneous fields, this *ab initio* approach is exact and works for arbitrary field construction.

In the following we will briefly review this method and describe how we deal with the model.

The time evolution of the Heisenberg field operator 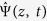 is given by the Dirac equation^14^,^30^.


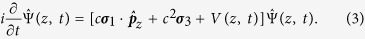


As in equation (2), the discussion is confined in one dimension. The field operator can be expressed in terms of the electron annihilation and positron creation operators as^26^


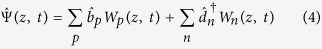



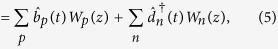


in which *p* and *n* denote the momenta of positive and negative energy states, *W*_*p*(*n*)_(*z*) = 〈*z*|*p*(*n*)〉 are solutions of the filed-free Dirac Hamiltonian (*V*(*z*, *t*) = 0), and ∑_*p*(*n*)_ denotes summation over all states with positive (negative) energy. The eigenstates of the filed-free Hamiltonian are


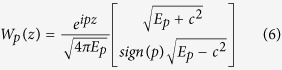



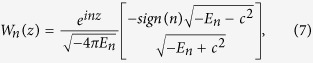


where 
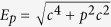
, and 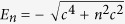 respectively. The time dependent single particle wave function *W*_*p*(*n*)_(*z*, *t*) can be got by introducing the time-evolution operator 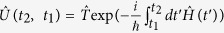,


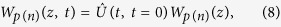


where 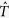 denotes the Dyson time ordering operator. In this paper, we use the numerical split operator technique^37^,^38^, then


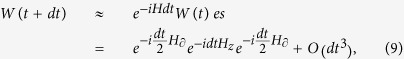


with


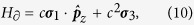



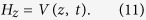


Practically, since the derivation (the momentum operator) can be implemented by replacing the operator 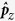 with its value *k*_*z*_ in momentum space, the evolution operation has the following form,


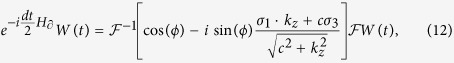



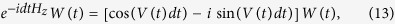


where 
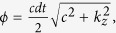
 and 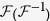 denotes Fourier transformation (inverse Fourier transformation).

Then, after the time dependent field operator 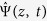 can be calculated, the number and the spacial distribution of electrons created from the vacuum (defined as 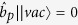, 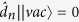) are obtained from the positive part of the field operator,


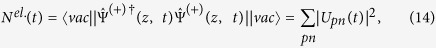



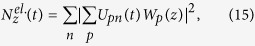


where 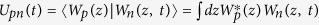. The pair number *N*(*t*) is equal to the electron number *N*^*el*.^(*t*).

The spacial distribution of the created positrons can be written as


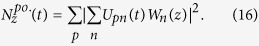


The positron number *N*^*po*.^(*t*) is equal to the electron number *N*^*el*.^(*t*). We can also get it from the negative part of the field operator by computing the number and spacial distribution of the holes. In this paper we use this expression, equation (16), to reduce the computational cost, because *U*_*pn*_ has been calculated in equation (14).

Furthermore, we can neglect the larger part of the momenta (when 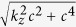 is far greater than *V* and *ω*), for its contribution to the matrix element *U*_*pn*_(*t*) is very small. In this paper the number of spatial points is 2048, and we only take 1024 discrete momenta in the evolution.

Based on the projection of the field operator onto the field-free electronic states in this method, *N*^*el*.(*po*.)^(*t*) here is actually the pair number if the field is turned off abruptly at time *t*. In this paper we present physical quantities for all time but focus on the moments when the field is absent.

## Additional Information

**How to cite this article**: Wang, Q. *et al.* Pumping Electron-Positron Pairs from a Well Potential. *Sci. Rep.* **6**, 25292; doi: 10.1038/srep25292 (2016).
